# Regional Image Features Model for Automatic Classification between Normal and Glaucoma in Fundus and Scanning Laser Ophthalmoscopy (SLO) Images

**DOI:** 10.1007/s10916-016-0482-9

**Published:** 2016-04-16

**Authors:** Muhammad Salman Haleem, Liangxiu Han, Jano van Hemert, Alan Fleming, Louis R. Pasquale, Paolo S. Silva, Brian J. Song, Lloyd Paul Aiello

**Affiliations:** 1 Manchester Metropolitan University, School of Computing, Mathematics and Digital Technology, Manchester, M1 5GD UK; 2 Optos, plc, Queensferry House, Carnegie Business Campus, Enterprise Way, Dunfermline, KY11 8GR Scotland UK; 3 Massachusetts Eye and Ear Infirmary, Harvard Medical School, Department of Ophthalmology, Boston, MA USA; 4 Beetham Eye Institute, Joslin Diabetes Center, Harvard Medical School, Department of Ophthalmology, Boston, MA USA

**Keywords:** Image processing and analysis, Machine learning, Computer-aided diagnosis, Glaucoma, Fundus camera, Scanning laser ophthalmoscope

## Abstract

Glaucoma is one of the leading causes of blindness worldwide. There is no cure for glaucoma but detection at its earliest stage and subsequent treatment can aid patients to prevent blindness. Currently, optic disc and retinal imaging facilitates glaucoma detection but this method requires manual post-imaging modifications that are time-consuming and subjective to image assessment by human observers. Therefore, it is necessary to automate this process. In this work, we have first proposed a novel computer aided approach for automatic glaucoma detection based on Regional Image Features Model (RIFM) which can automatically perform classification between normal and glaucoma images on the basis of regional information. Different from all the existing methods, our approach can extract both geometric (e.g. morphometric properties) and non-geometric based properties (e.g. pixel appearance/intensity values, texture) from images and significantly increase the classification performance. Our proposed approach consists of three new major contributions including *automatic localisation of optic disc*, *automatic segmentation of disc*, and *classification between normal and glaucoma based on geometric and non-geometric properties of different regions of an image*. We have compared our method with existing approaches and tested it on both fundus and Scanning laser ophthalmoscopy (SLO) images. The experimental results show that our proposed approach outperforms the state-of-the-art approaches using either geometric or non-geometric properties. The overall glaucoma classification accuracy for fundus images is 94.4 % and accuracy of detection of suspicion of glaucoma in SLO images is 93.9 %.

## Background

Glaucoma is one of the leading causes of irreversible blindness worldwide accounting for as much as 13 % of all cases of vision loss [1, 2]. It is estimated that more than 500,000 people suffer from glaucoma in England alone, with more than 70 million people affected across the world [3, 4]. The changes occur primarily in the optic disc [5], which gradually can lead to blindness if left untreated. As glaucoma-related vision loss is irreversible, early detection and subsequent treatment are essential for affected patients to preserve their vision. Conventionally, retinal and optic nerve disease identification techniques are based in part, on subjective visual assessment of structural features known to correlate with the pathologic disease. When evaluating retinal images, optometrists and ophthalmologists often rely on manual image enhancements such as adjusting contrast and brightness and increasing magnification to accurately interpret these images and diagnose results based on their own experience and domain knowledge. This process is time consuming and its subjective nature makes it prone to significant variability. With the advancement in digital imaging techniques, digital retinal imaging has become a promising approach that leverages technology to identify patients with glaucoma [6]. Retinal imaging modalities such as fundus cameras or Scanning Laser Ophthalmoscopes (SLO) have been widely used by the eye clinicians. Retinal imaging with automated or semi-automated image analysis algorithms can potentially reduce the time needed by clinicians to evaluate the image and allow more patients to be evaluated in a more consistent and time efficient manner [7, 8].

**Fig. 1:**
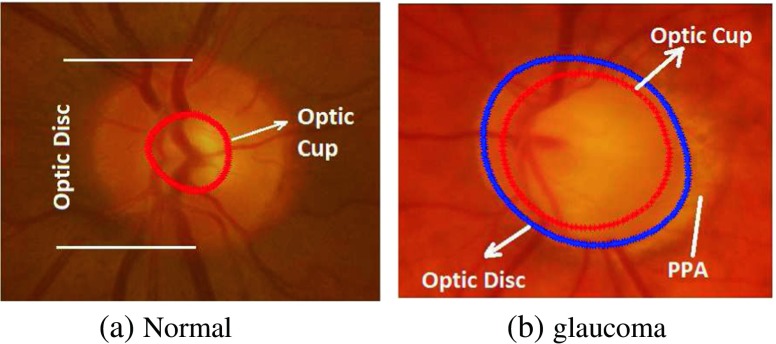
Comparison of the optic disc area of the **a** normal and **b** glaucomatous image. The cup boundary is shown with the red outline in both images and disc boundary is shown with blue outline in **(b)** only. There is significantly larger cup in relation to the size of the optic disc in the glaucoma image compared to the normal image. Inferior sector Peripapillary Atrophy (PPA) in the glaucoma image **(b)** is also evident possibly due to concomitant erosion of the inferior neuro-retinal rim tissue

Glaucoma is associated with erosion of the neuroretinal rim which often enhances the visibility of chorioretinal atrophy in the peripapillary tissue (the latter is referred to as peripapillary atrophy (PPA)) [9, 10]. This can be quantified by geometrical measures (e.g. an increased cup-to-disc ratio (CDR), a well-established glaucoma indicator in the research community, particularly in the vertical meridian (Fig. 1).

There are several efforts made for the classification between normal and glaucomatous patients, which we can broadly divide into two categories including: geometrical based methods and non-geometrical based methods. The geometrical based methods involve the automatic calculation of glaucoma associated geometrical features (e.g. optic cup, disc shapes/diameters , or CDR). Their automatic determination require automatic segmentation of anatomical structures such as optic disc and optic cup in a retinal image. Nayak et al. [11] performed segmentation using morphological operations [12] for calculation of the CDR and performed classification using neural networks. The classification accuracy was 90 % on 15 images after training the classifier on 46 normal and glaucoma images. Other efforts stated the accuracy of the methods in terms of optic disc and cup segmentation [13].

On the other hand, the non-geometrical based methods extract image features such as pixel appearance, textural properties, intensity values, colour, etc. of the optic disc cropped image. Bock et al. [14] calculated image texture, Fast Fourier Transform (FFT) Coefficients, Histogram Models, B-Spline coefficients on the illumination corrected images. Based on these features they calculated a Glaucoma Risk Index using a two-stage classification scheme. Dua et al. [15] used Wavelet-Based Energy Features and compared the performance of different classifiers such as Support Vector Machines (SVM), Naive Bayes [12], Random Forests [16] and Sequential Minimal Optimization (SMO) [17]. Texture and Higher Order Spectra (HOS) based information has also been used for the classification between normal and glaucoma images [18, 19]. Here different classifiers were investigated and it was found the maximum accuracy was achieved using the SVM. All these methods focus on image features of the retinal image obtained from 45 ^∘^ field of view fundus camera except the Bock’ method which focus on optic disc cropped image.

### Hypothesis and contributions

Despite the existing methods mentioned above are encouraging, they only focus on either geometrical properties or non-geometrical properties. In fact, in an optic disc cropped image, there are certain indications in different regions of optic disc cropped image apart from increased cup size (such as PPA) which can be quantified for automatic glaucoma classification. Our **hypothesis** is that the classification between normal and glaucoma images can be improved through combining both geometrical and non-geometrical features.

Therefore, different from all the existing approaches, we propose a novel holistic approach: *Regional Image Features Model* (RIFM) to extract both geometrical and non-geometrical properties in different regions of optic disc and its surroundings. The proposed approach can automatically, accurately localise, segment the optic disc, divide optic disc cropped images into different regions, and classify an image into the right category (i.e. normal or glaucoma). Our contributions include:

1. A new accurate algorithm of automatic optic disc localisation based on *weighted feature maps* to enhance optic disc and vasculature converging at its centre.
2. A new accurate, automatic optic disc segmentation method derived from our previous work [20] so as to avoid misguidance due to vasculature or atrophy in case of glaucoma.
3. A new regional image feature model (RIFM) which can extract both geometrical and non-geometrical features from different regions of optic disc and its surroundings.

The rationale behind the RIFM lies in automatic localisation and segmentation of optic disc and then dividing its surrounding into five regions: the optic disc area, inferior (I), superior(S), nasal(N) and temporal(T). In clinical practice, clinicians often visually inspect these regions and make a diagnosis. There is currently no existing work on automation of this process. Based on different regions, the features including textural, frequency, gradient, colour and illumination information are then extracted. The classifier is then built for classification between glaucoma and non-glaucoma images.

We have compared our method against the existing approaches and evaluated our prototype on both fundus and Scanning Laser Opthalmoscope (SLO) images obtained from our collaborator, Optos [21]. To the best of our knowledge, this is the first approach with a combination of geometric and non-geometric properties, which can automatically divide regions based on clinical knowledge and perform classification between normal and glaucoma, applicable to both fundus and SLO images.

The rest of this paper is organised as follows: “??” introduces datasets used in our experiments. “Method” discusses our proposed method. Section “Experimental evaluation and discussion” provides the quantitative and visual results of our proposed method. Section “Conclusion” summarizes and concludes the proposed work.

## Datasets used for experimentation

### RIM-ONE

RIM-ONE (An Open Retinal Image Database for Optic Nerve Evaluation) [22, 23] is a fundus image dataset composed of 85 normal and 39 glaucoma images. All the images have been annotated with boundaries of optic disc and optic cup from which we calculated vertical CDR values. The retinal images in the dataset were acquired from three different hospitals located in different regions of Spain. They have compiled the images from different medical sources which guarantee the acquisition of a representative and heterogeneous image set. All the images are non mydriatic retinal photographs captured with specific flash intensities, thus avoiding saturation.

### SLO images

All ultrawide field SLO images were obtained using the Optos P200MA [21]. Unlike traditional flash-based fundus cameras, this device is able to capture a single wide retinal image without dilation. The image has two channels: red and green. The green channel (wavelength: 532nm) provides information about the sensory retina to retinal pigment epithelium whereas the red channel (wavelengh: 633nm) shows deeper structures of the retina towards the choroid. Each image has a dimension of 3900×3072 and each pixel is represented by 8-bit on both red and green channels. The SLO images have been taken from 19 patients suspected with glaucoma while 46 images are from non-glaucomatous patients. The images have been annotated and graded by glaucoma specialists at Harvard Medical School, Boston, MA, USA. The annotations are provided in terms of boundaries of optic disc and optic cup as well as vertical CDR values.

## Method

**Fig. 2:**
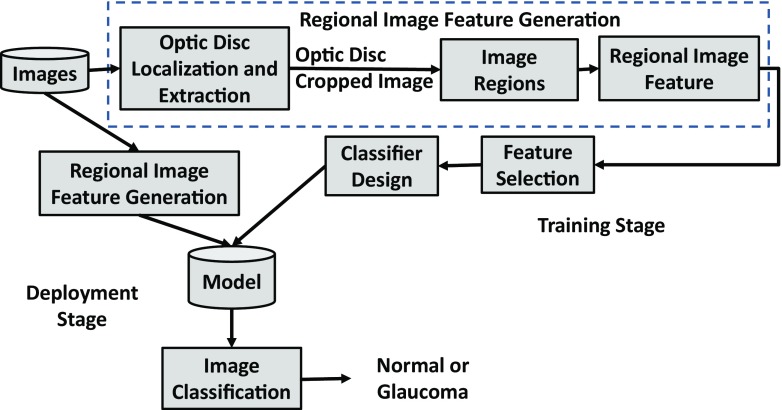
Block diagram of regional image features model

The block diagram of the RIFM is shown in Fig. 2. We first localise and segment the optic disc from an image. Then the image will be divided into different regions. The main rationale of dividing the image into different regions is that geometrical changes in glaucoma can have different image features compared to normal images. For example, the higher CDR will result in higher intensity values in the optic disc in cases of glaucoma. Also the occurrence of atrophy due to glaucoma will result in different texture around optic disc surroundings. The deployment stage classifies the test image between normal and glaucoma. The subtasks of the block diagram are discussed in the following subsections.

### Automatic localisation of optic disc

**Fig. 3:**
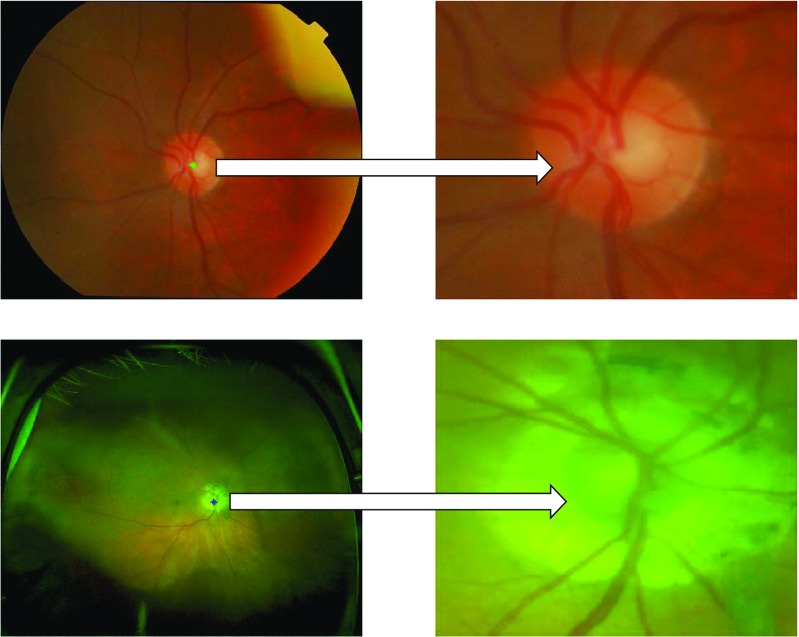
Examples of optic disc localization on fundus image (*first row*) and SLO image (*second row*). The SLO image optic disc has been affected by atrophy area around but our proposed optic disc localization was able to locate it accurately

Although the optic disc is often the brightest region in the retinal scan, its localisation can be misguided due to the presence of disease lesions, instrument reflections and the presence of PPA. Therefore, optic disc localisation can be more accurate by determining retinal vasculature convergence point which converges at the centre of the optic disc [24]. However, vasculature area are not clearly visible in the cases with high instrument reflection from PPA. In order to make optic disc localisation more robust, we have developed a new localisation method as shown in **Algorithm 1** which involves development of two *weighted feature maps* for enhancing the optic disc (*F*_1_) and vasculature structure (*F*_2_). The equations of the feature maps we developed are shown in Eq. 1. Although the summation of x and y gradients can be helpful in determining bright regions like optic disc on *Y* (intensity map in *YUV* colour space [25]), the Fast Radial Symmetry Transform [26] with specified radius *r* will enhance the optic disc further compared to other bright regions. Similarly, matched filtering [27] will enhance the vasculature structure further on the intensity map. In matched filtering, the mean Gaussian response in different directions (1) with difference of 30 ^∘^ among adjacent *𝜃* values is taken. *σ* is set to 4 for fundus images and 1.5 for SLO images as SLO images have low resolution optic disc due to its wide FOV. The mean response of matched filter is *min-max* normalized in order to make the response consistent for every image. For optic disc localisation we have performed the exhaustive search in *F*_2_ horizontally and in *F*_1_ vertically. The Eq. 2 estimates the optic disc centre by exhaustive search. Due to resolution difference, the maximum dimensions of optic disc are 400 and 150 in case of fundus and SLO images whereas the maximum vessel width is 50 and 12 respectively. The examples of optic disc localization in fundus and SLO images are shown in Fig. 3.

**Figure:**
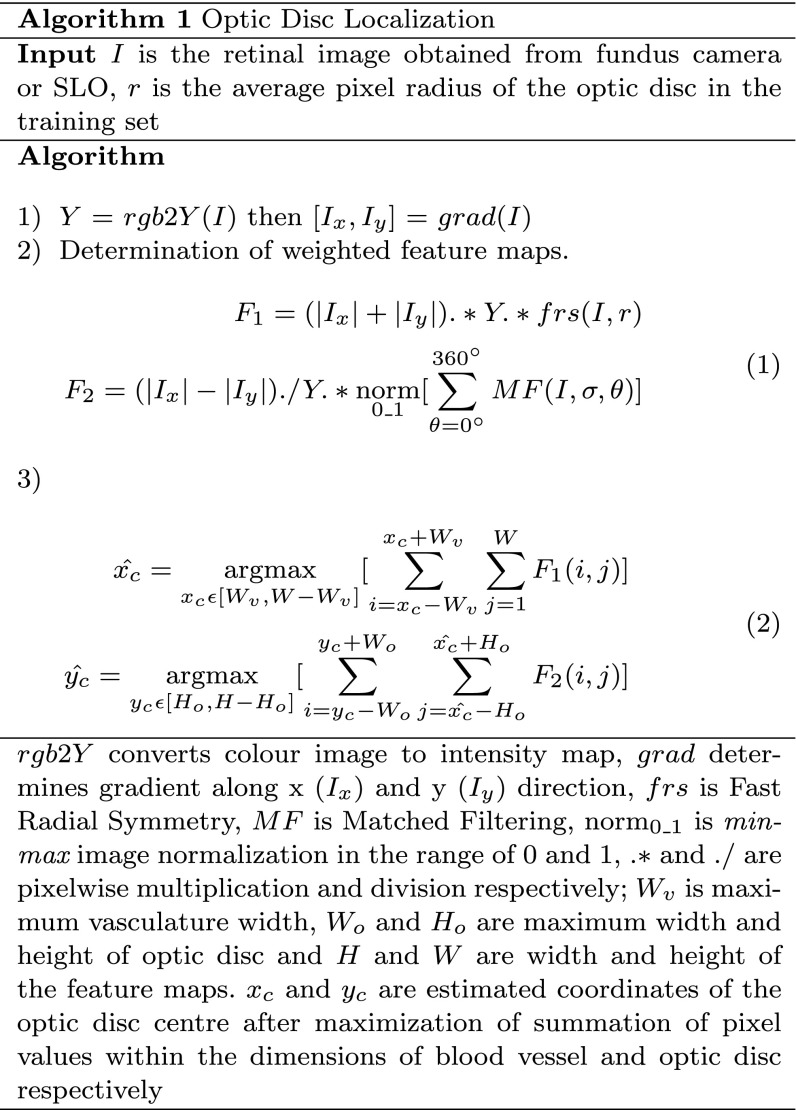

[!h]

### Automatic segmentation of optic disc

#### The segmentation algorithm

After optic disc localisation, the next step is its segmentation. Building upon our proposed work, instead of determining optic disc contour on the gradient map [20], we have developed the feature maps and then estimated the contour by minimizing the distance between normal profiles of feature maps from each contour point in a test image and mean of the images in the training set. These feature maps have been determined by image convolution with a Gaussian filter bank [28]. Convolving the image with a Gaussian filter bank can determine the image features at different resolutions. The Gaussian filter can be given as:

3 $$ \mathcal{N}(\sigma,i,j) = \frac{1}{2\pi\sigma^{2}}e^{-\frac{i^{2} + j^{2}}{2\sigma^{2}}} $$

**Fig. 4:**
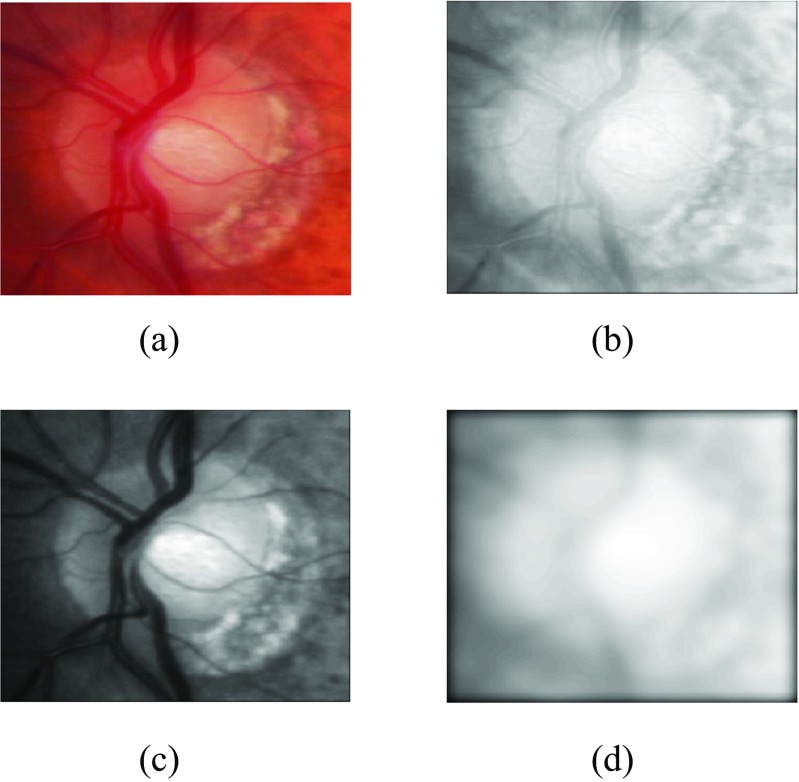
Elaboration of optic disc image after image convolution with a Gaussian filter with **a** original image, **b** *red* channel convolution at *σ*=2, **c** *green* channel convolution at *σ*=2 and **d** *red* channel convolution at *σ*=8

Convolving the retinal image with a Gaussian filter bank at different scales *σ* determines the image details at different resolutions by adding the blur while increasing the scale. The Gaussian filter bank includes Gaussian $\mathcal {N}(\sigma )$, its two first order derivatives $\mathcal {N}_{x}(\sigma )$ and $\mathcal {N}_{y}(\sigma )$ and three second order derivatives $\mathcal {N}_{xx}(\sigma )$, $\mathcal {N}_{xy}(\sigma )$ and $\mathcal {N}_{yy}(\sigma )$ in horizontal(x) and vertical(y) directions. The retinal images have been convolved at different scales *σ*=2,4,8,16 as PPA has been diminished at higher scale whereas optic disc edges are more visible at lower scales (Fig. 4). Morever, the image convolution has been performed at both red and green channels as the optic disc boundary has more meaningful representation without PPA or vasculature occlusion at *σ*=8 but PPA is more visible at green channel at *σ*=2 which can be helpful while training the features inside and outside the optic disc boundary. Before calculation of features maps, we have performed vasculature segmentation [29] followed by morphological closing in 8 directions and retaining maximum response for each vessel pixel. This is to avoid misguidance due to vasculature occlusion.

We have then evaluated the profiles from the line normal to each contour point from the feature maps and calculated the mean *V*_*train*_ across the images in the training set. The length of the normal lines can be set as discussed in [20]. We then estimate new contour $\hat {\textbf {Y}}$. For test profile *V*, each of the contour point *n* can be achieved by Eq. 5 in **Algorithm 2** where *M* is the number of feature maps. Among *P* test profiles, the optimum profile can be estimated with minimum mahalanobis distance [30] with *V*_*train*_. Then we applied the statistical shape modeling so as to adjust the estimated contour **Y** with the mean of shapes in the training set. This has significantly increased the segmentation performance. The optic disc boundary can now be represented as *X*. After determining optic disc boundary model, we have readjusted the optic disc centre as:

4 $$ x_{c} = \frac{{\sum}_{i=1}^{N} X_{x}(i)}{N} \, \, y_{c} = \frac{{\sum}_{i=1}^{N} X_{y}(i)}{N} $$

where *X*_*x*_ and *X*_*y*_ are positions of *X* in x-axis and y-axis respectively.

**Figure:**
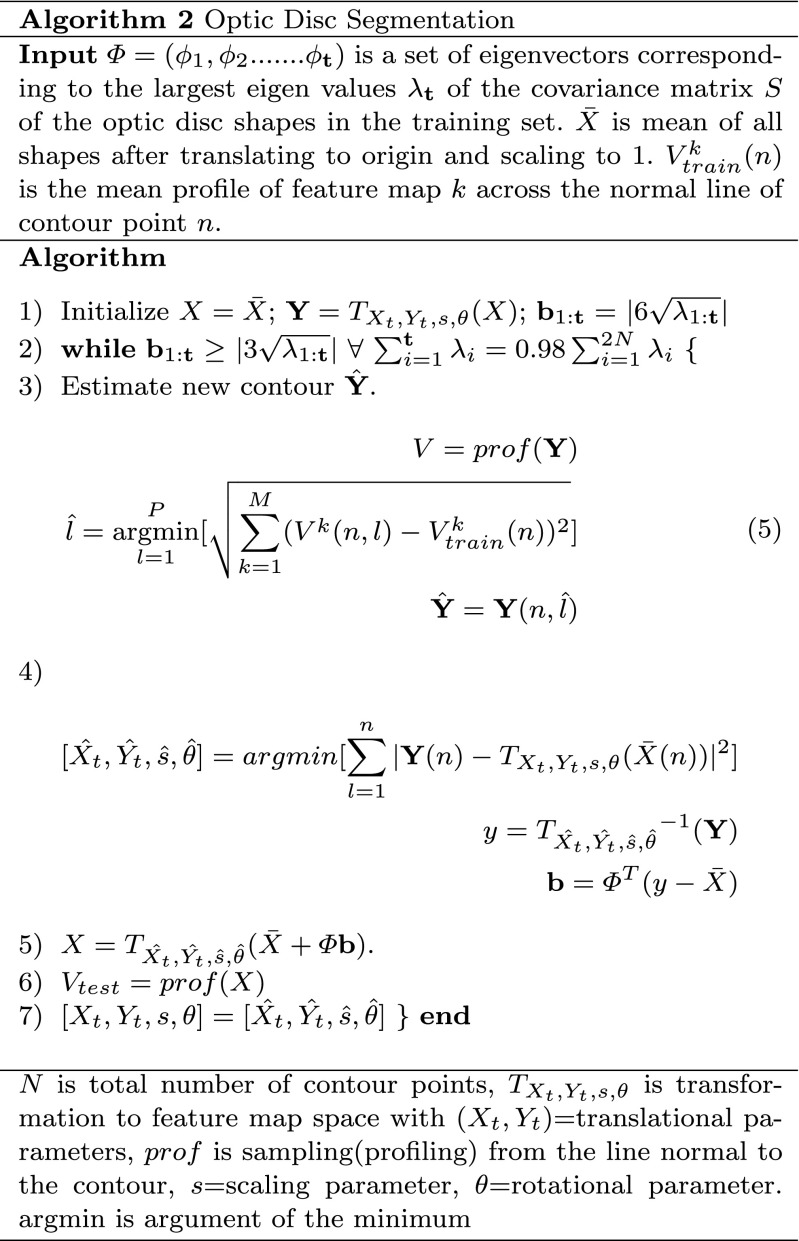

### Regional image features model

After optic disc segmentation, we need to determine *Regional Image Feature Model* (RIFM). Here we have performed the following steps:

#### Determination of regions in the optic disc cropped image

**Fig. 5:**
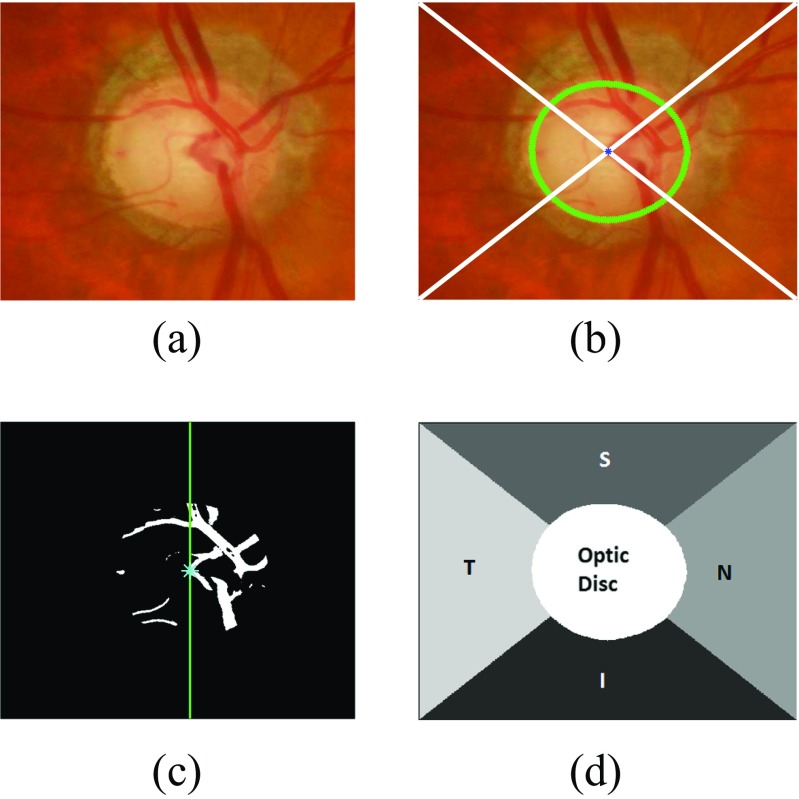
Different regions of the optic disc centred image with **a** image of a *right* eye **b** image divided into different quadrants with the optic disc boundary represented with *green* and centroid with *blue* colour, **c** vasculature area within the optic disc with higher area on the right side **d** optic disc centred image divided into different regions

i. Optic disc cropping which should be twice the maximum diameter of optic disc in the dataset as shown in Fig. 5a. This has been done in order to fully include atrophy area around optic disc and other features if present.
ii. Connecting the optic disc centre (*x*_*c*_,*y*_*c*_) to each corner of the cropped image. This divides the image into 4 different quadrants shown in Fig. 5b.
iii. Naming the regions as inferior(I), superior(S), nasal (N) and temporal(T) regions. I and S regions are fixed for each image. However, N and T regions can be named after determining if the image is from left eye or right eye. The algorithm calculates the vasculature area (segmented during optic disc segmentation) within the optic disc in both halves of the image. In this case, the vasculature area is higher on the right so this image is considered as right eye image (Fig. 5c). Therefore, N and T regions will be on right side and left side respectively (Fig. 5d).
iv. Generating the image regions mask representing optic disc and different regions in its surroundings (Fig. 5d).

#### Determination of regional image features

**Fig. 6:**
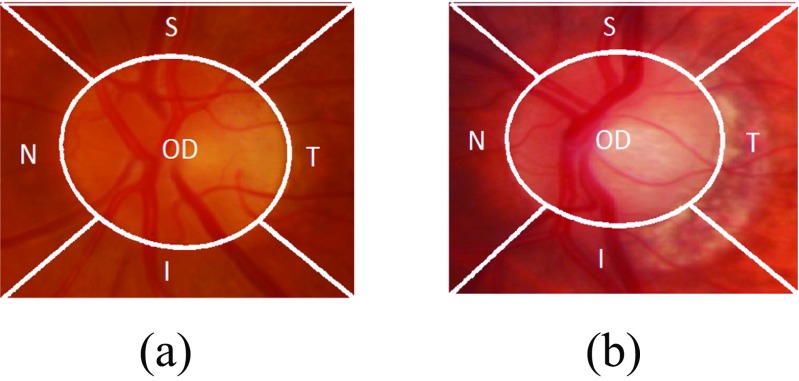
Comparison of **a** normal and **b** glaucoma images after division of optic disc cropped image into different regions

After the generation of different regions in the optic disc cropped image, the next step is to determine the image-based features for each region. Apart from geometrical features (e.g. optic disc size, CDR), there are certain representations of an image which can distinguish two images taken from different states. These representation can be quantified by calculating features representing the image. In our case, these different states are normal and glaucoma. After optic disc segmentation and dividing the image into different regions, we can then analyze each region separately which can lead to unique contributions from each region in determining glaucoma classification. The features calculated for each region will represent a different column in a feature vector. Consider the examples from each of normal and glaucoma in Fig. 6. Both examples have been taken after optic disc segmentation and division of image into different regions. The boundary of the optic cup is not clearly evident in either image, which can lead to misidentification of CDR. Also the presence of PPA in the I and T regions in the glaucoma image is not sufficient to make a diagnosis of glaucoma [10]. Therefore, we need to evaluate the difference between normal and glaucoma by calculating the features which can represent texture, spatial and frequency based information.

For each region, we have generated the feature matrix on the basis of different features as follows:

6 $$ FM \,=\, \left[ \begin{array}{c c c c c c} \!F\!M_{RG}^{\textit{dg}}\! &\! F\!M_{RG}^{\textit{texoff}} & F\!M_{RG}^{\textit{texscale}} & F\!M_{RG}^{\textit{g}} F\!M_{RG}^{\textit{gab}} & F\!M_{RG}^{\textit{wav}} \end{array} \right] $$

where *RG* represents red and green channel respectively, *texoff* represents textural features with variable offset values, *texscale* represent textural features with variable scale, *dg*, *g*, *wav* and *gab* represent dyadic Gaussian, gradient features, wavelet features and gabor filter features. The details of each feature type are described below:

##### Gaussian features

The mean value of each region after convolving the image with each Gaussian filter and its first and second order derivatives determined for optic disc segmentation has been calculated to generate $FM_{RG}^{\textit {g}}$. We have 6 gaussian filters convolved at scales *σ*=2,4,8,16 for red and green channels and region which makes the length of $FM_{RG}^{\textit {g}}$ equal to 240.

##### Textural features

Textural features can be determined by evaluating Grey Level Co-occurrence Matrix (GLCM) [31, 32]. GLCM determines how often a pixel of a grey scale value *i* occurs adjacent to a pixel of the value *j*. The pixel adjacency can be observed in four different angles i.e. *𝜃*=0^∘^,45^∘^,90^∘^,135^∘^. For the region of size *p* x *q*, we perform second order textural analysis by constructing the GLCM (*C*_*d*_(*i*,*j*)) and probability of pixel adjacency (*P*_*d*_(*i*,*j*)) as:

7 $$ \begin{array}{llll} C_{d}(i,j)=\left\{\begin{array}{llll} (p,q), (p+{\Delta} x,q + {\Delta} y):\\ I(p,q) = i,I(p+{\Delta} x,q + {\Delta} y)=j \end{array}\right. \\ P_{d}(i,j) = \frac{C_{d}(i,j)}{{\sum}_{i}{\sum}_{j} C_{d}(i,j)} \end{array} $$

where Δ*x* and Δ*y* are offset values. The dimensions *p* and *q* represent the bounding box of the particular region among I,S,N,T and OD. We have evaluated 20 features representing textural information which are enlisted in Table 10 [33]. Since we have significantly large size of regions, the pixel adjacency can be evaluated at different offset values Δ*x* and Δ*y*. We have varied these values ranging from 1 to 10 for both Δ*x* and Δ*y* to generate $FM_{RG}^{\textit {texoff}}$. We have evaluated 20 textural features for each red and green channel (blue channel is set to zero in SLO so we did not calculated for fundus images as well).

Apart from varying the offset values, we have also calculated these features by convolving the image with Gaussian filter at different scales *σ*=2,4,8,16 after fixing offset values at 1 for generating $FM_{RG}^{\textit {texscale}}$.

##### Dyadic gaussian features

**Fig. 7:**
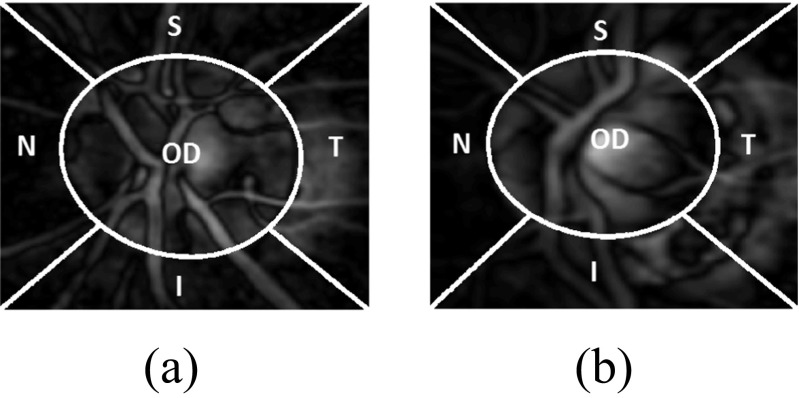
Comparison of **a** normal and **b** glaucoma images of Fig. 6 at *Y*_*rg*_(2,5)

The Dyadic Gaussian features involve the downsampling of the optic disc cropped image at multiple spatial scales [34, 35]. The calculation of absolute difference at different spatial scales can lead to the development of low-level visual ‘feature channels’ which can discriminate between normal and glaucoma images. We can generate certain features from red channel, green channel and combinations of both channels. The blue channel is set to zero for SLO therefore we do not take this into account for fundus images as well. Apart from Red(R) and Green(G) channels, we have determined the feature channels as follows:

8 $$ \begin{array}{lllll} I_{mn} = \frac{(R+G)}{2} \\ Y_{rg} = R+G-2|R-G|\\ \end{array} $$

where *I*_*mn*_ is the mean response of the both channels and the *Y*_*rg*_ shows their mixed response i.e. yellow channel. The absolute difference of the particular feature channels at different spatial scales lead to determination of excitation and inhibition response. For determination of excitation and inhibition response, we have centre levels *c* and surround levels *s* of the spatial scales respectively. This can be calculated as:

9 $$ \begin{array}{llll} I_{mn}(c,s) = |I_{mn}(c)-Interp_{s-c}I_{mn}(s)| \\ RG(c,s) = |(R(c)-G(c))-Interp_{s-c}(R(s)-G(s))| \\ Y_{rg}(c,s) = |(Y_{rg}(c))-Interp_{s-c}(Y_{rg}(s))| \\ \end{array} $$

where *I* *n* *t* *e* *r* *p*_*s*−*c*_ represent interpolation to *s*−*c* level. Note that *s* = *c* + *d*. If we calculate mean response of each region i.e. $FM_{RG}^{\textit {dg}}$=[I(c,s),RG(c,s),BY(c,s)]:

10 $$ \begin{array}{llll} I^{reg}(c,s) = {\sum\limits_{i}^{N}} \frac{I(c,s,n)}{N} \\ RG^{reg}(c,s) = {\sum\limits_{i}^{N}} \frac{RG(c,s,n)}{N} \\ BY^{reg}(c,s) = {\sum\limits_{i}^{N}} \frac{BY(c,s,n)}{N} \end{array} $$

where *N* is number of pixels in the region. The dyadic Gaussian features can excite the optic disc region while inhibiting the regions in its surroundings. For the case of glaucoma, the excited optic disc region can have higher intensity values due large optic cup size while inhibition of atrophy area in its I and T regions can also contribute towards glaucoma classification as shown in Fig. 7. We have calculated dyadic Gaussian features at *c* and *d*=[2,3,4] which make *c*,*s* pairs as [2-4,2-5,2-6,3-5,3-6,3-7,4-6,4-7,4-8]. In this way we have 135 features generated from dyadic Gaussian as shown in Table 1.

**Table 1:** Number of features from each feature type

Feature types	Number of regional features generated	Number of global features
$FM_{RG}^{\textit {g}}$	6 filters * 4 scales * 2 channels * 5 regions = 240	48
$FM_{RG}^{\textit {texoff}}$	20 * 10 offset values * 2 channels * 5 regions = 2000	400
$FM_{RG}^{\textit {texscale}}$	20 * 4 scales * 2 channels * 5 regions = 800	160
$FM_{RG}^{\textit {dg}}$	9 pairs * 3 channels * 5 regions = 135	27
$FM_{RG}^{\textit {gab}}$	4 scales * 5 gamma * 7 frequencies * 4 orientations * 2 channels * 5 regions = 5600	1120
$FM_{RG}^{\textit {wav}}$	5 families * 4 bands * 2 channels * 2 types * 5 regions = 400	80
**Total**	9175	1835

##### Gabor features

Gabor filters can be convolved with the image at different frequencies and orientations which can generate different feature channels for image classification [36]. For determining $FM_{RG}^{\textit {gab}}$, we have taken mean response of the gabor filter in the region. The gabor filter is represented as:

11 $$ Gb(x,y,\theta,f,\sigma,\gamma) = \exp(-\frac{1}{2}(\frac{{\hat{x}^{2}}}{\sigma^{2}}+\frac{\hat{y}^{2}\gamma^{2}}{\sigma^{2}})*\exp(i2\pi fx) $$

12 $$ \hat{x} = x cos\theta + y sin\theta \qquad \hat{y} = y cos\theta - x sin\theta $$

*x* and *y* are image pixel coordinates. Here we have varied *σ*=[2,4,8,16], *γ*=[$\frac {1}{3},\frac {1}{2}$,1,2,3], *f*=[$\frac {1}{4},\frac {1}{3},\frac {1}{2}$,1,2,3,4], *𝜃*=[0^∘^,45^∘^,90^∘^,135^∘^]. The *γ* value is varied so as to determine the responses when scale of x and y axis are equal and unequal at different scales. On the similar grounds, frequency is varied in such a way so as to determine the response when wavelength is higher than frequency and vice versa. In this way a total number of 5600 features have been determined with different combination of gabor parameters.

##### Wavelet features

We have calculated Discrete Wavelet Transform (DWT) features $FM_{RG}^{\textit {wav}}$ denoted by *ψ* [37]. The DWT features captures both spatial and frequency information of the image. DWT analyses the image by decomposing it into a coarse approximation via low-pass filtering and into detail information via high-pass filtering. Such decomposition is performed recursively on low-pass approximation coefficients obtained at each level [38]. The image is divided into four bands i.e. A(Top left (LL)), H (Top Right (LH)), V(Bottom Left (HL)) and D(Bottom Right (HH)). As an example, LH indicates that rows and columns are filtered with low pass and high pass filters, respectively. DWT decomposition is calculated on five different wavelet families i.e. haar, db3, rbio3.3, rbio3.5, rbio3.7. For a particular region in the optic disc cropped image, we can calculate two types of features using these bands i.e. average value of the coefficients (*ψ*_*Avg*_) and energy of the coefficients (*ψ*_*Energy*_). As an example, the average value and average energy of D band are derived from the wavelet coefficients, as shown below;

13 $$ \begin{array}{lllll} {\psi^{D}}_{Avg} = \frac{1}{p\,q}\sum\limits_{i={p}}\sum\limits_{j={q}}|D_{band}(i,j)| \\ {\psi^{D}}_{Energy} = \frac{1}{p^{2}\,q^{2}}\sum\limits_{i={p}}{\sum}_{j={q}}(D_{band}(i,j))^{2} \end{array} $$

where *p* and *q* represents width and height in pixels of the region respectively. We have performed the DWT decomposition to only one level as features calculated for higher levels were not significant (*p*_*value*_≥0.05) thus they were not included in the feature set.

#### Z-score normalization

After determination of feature matrix, the feature matrix is normalized using z-score normalization [39]. It can be represented as:

14 $$ FM_{ZS} = \frac{FM-\mu_{f}}{\sigma_{f}} $$

where *μ*_*f*_ is the mean of the features and *σ*_*f*_ is the standard deviation across the examples in the training set.

#### Feature selection

**Fig. 8:**
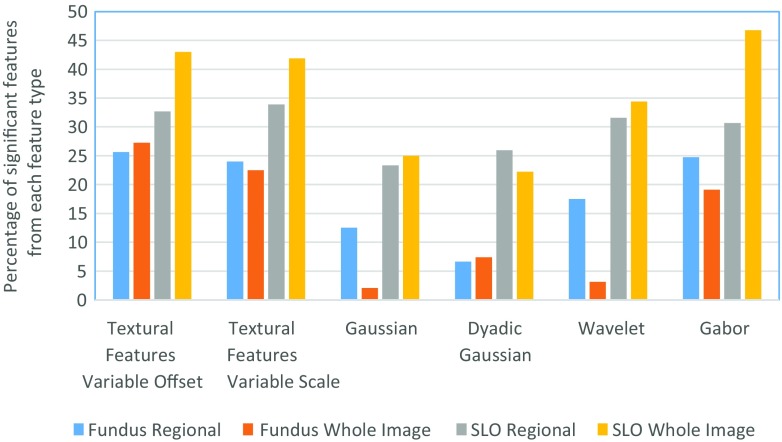
Percentage of significant features selected from each category

Due to division of optic disc cropped images into five regions, the number of features generated is five times larger than the situations where features are generated for a whole image. Since the determination of classifier constructed on such a high dimension of features is not computationally efficient and also some of these features may lead to depreciation of classifier performance, we have selected the features based on *p*_*value*_ which are statistically significant (*p*_*value*_≤0.05) towards glaucoma classification. Table 1 shows the number of features generated by each feature type whereas the percentage of features selected from each feature type has been shown in Fig. 8. For fundus images, 2201 regional features out of 9175 features have been significant towards glaucoma classification and 2836 regional features have been significant towards classification in SLO images. The bar plot shows that the textural and gabor features can be more clinically significant compared to other types of features. Figure 8 and Table 1 also provides information regarding the total features generated and number of significant features selected for global features (whole image features) for comparison purpose.

After selection of relevant features, the feature dimension is still high for classifier construction. In order to select features most relevant towards classification, we have performed feature selection on significant feature set. In our case, we have adopted wrapper feature selection [40]. The wrapper feature selection is an iterative procedure of maximizing classification performance. In the feature selection procedure, initially the data is divided into *k* folds (in our case *k*=5). Then the first feature is selected which has maximum mean classification performance across the folds. During the next iterations, the features together with previously selected features result in highest mean classification performance are selected. This process continues until there is little or no maximization towards classification performance. This process is in contrast to the filter selection approach [41] in which the feature ranking is performed according to individual evaluation performance of each feature. The individual evaluation performance is quantified according to their classification power and the features beyond certain threshold value are selected for classifier construction. However, our recent study [33] has shown that features selected by wrapper feature selection procedure outperforms filter feature selection despite the fact that filter selection approach selects the best features from the pool whereas wrapper feature selection does not necessarily follow the similar approach. Nevertheless, the wrapper feature selection approach has been performed on the features which have been filtered out with *p*_*value*_≤0.05.

For quantification of classification performance of the wrapper feature selection, we have certain performance measures such as Area Under the Curve (AUC), linear classification accuracy and quadratic classification accuracy. The AUC can be quantified by determining the area under Receiving Operating Characteristics (ROC). ROC is a graphical plot that illustrates the performance of a binary classifier system by area under it as it is created by plotting the true positive rate against the false positive rate at various threshold settings [39]. The ROC curve of the selected regional image features has been shown in Fig. 12 with a red plot. The wrapper feature selection by maximizing AUC is termed as ‘wrapper-AUC’. On the other hand, linear classification accuracy is based on Linear Discriminant Analysis (LDA) by maximizing the distance between classes while minimizing the variance within each class. Quadratic Discriminant Analysis (QDA) works on similar principle as its linear counterpart except the classification boundary between classes is not linear and covariance matrix may not be identically equal for each class. The wrapper feature selection by maximizing LDA and QDA are termed as ‘wrapper-LDA’ and ‘wrapper-QDA’ respectively.

**Fig. 9:**
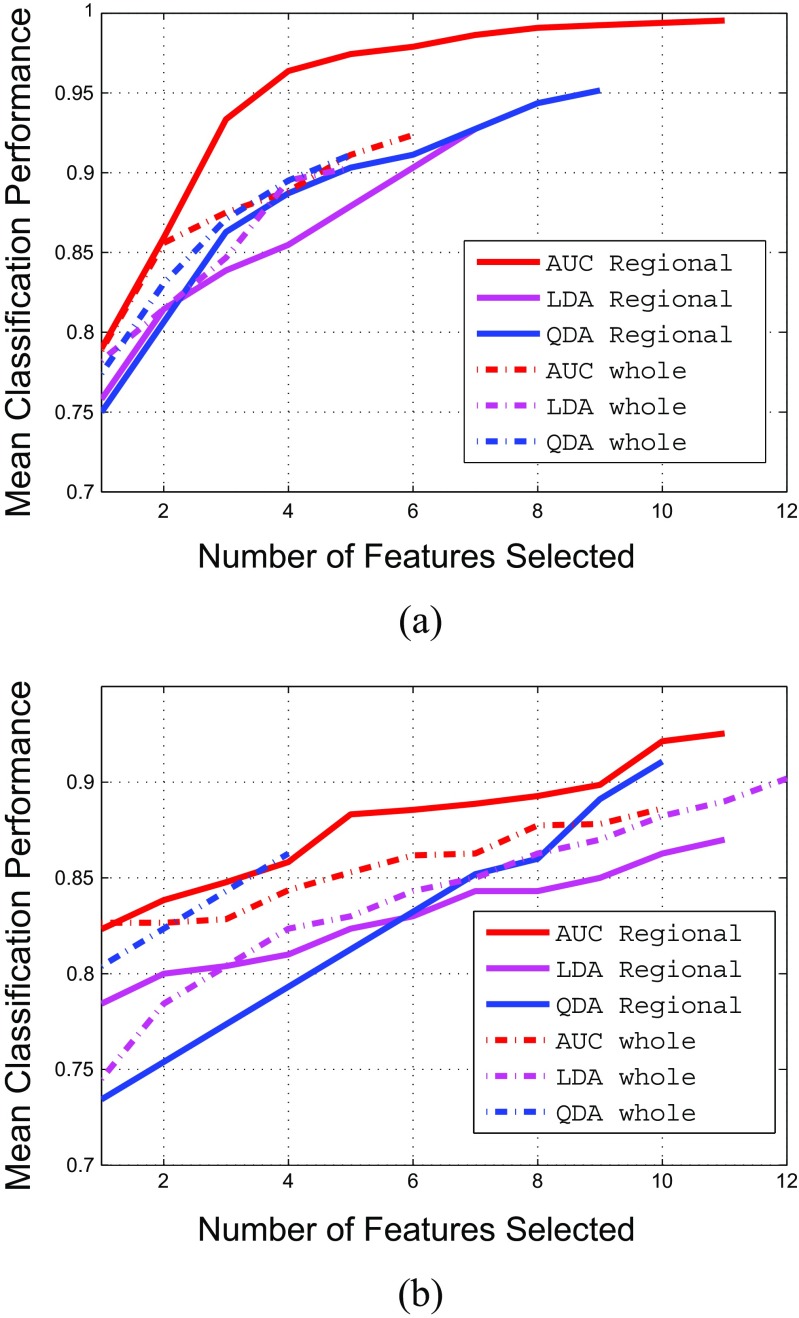
Feature selection procedure for both regional and whole image features in different classification performance

We have run the wrapper feature selection with the performance measures mentioned previously on the significant features. The number of features selected based on different feature selection methods (wrapper-AUC, wrapper-LDA and wrapper-QDA) is shown in Table 2. For example, for RIMONE dataset, when using wrapper-AUC, the total number of regional features selected is 11. The total number of feature selected for wrapper-LDA and wrapper-QDA is 7 and 9 respectively. The results of feature selection procedure have been shown in Fig. 9. The results shows that if the features are selected by AUC as performance measure of wrapper feature selection, we can achieve significantly higher classification accuracy compared to other performance measures. Also the classification power of regional features have been significantly better compared to global features both in case of fundus and SLO images. Moreover, the results in Table 2 shows that apart from the optic disc region, the other regions (such as I) can also play significant role in glaucoma classification. The list of features selected after wrapper feature selection for both fundus and SLO images have been shown in Table 3. The list has mostly been dominated by either textural or Gabor features. As a reference, $H_{diffG(2)}^{OD}$ is the ‘Difference Entropy’ from Table 10 where *OD* in superscript represent the optic disc region, *G* in subscript represent the green channel where as 2 in subscript represent the offset value. If the number is not in subscript (as in case of $corr_{G}^{OD}(2)$), then it represent the scale (*σ*) value.

**Table 2:** Comparison of number of features selected by each feature selection methods from different regions and total number of features selected

	RIMONE			SLO images		
Regions	Wrapper-AUC	Wrapper-LDA	Wrapper-QDA	Wrapper-AUC	Wrapper-LDA	Wrapper-QDA
I	4	1	3	4	3	3
S	1	4	3	1	3	2
N	0	0	1	2	5	3
T	2	0	1	2	0	2
OD	4	2	1	2	0	0
**Total**	11	7	9	11	11	10

**Table 3:** Symbols of features selected by sequential maximization approach. These features also represent the x-axis of Fig. 9

Criteria	Fundus image	SLO images
**Regional Features**		
AUC	$H_{diffG(2)}^{OD}$, $Y_{rg}^{I}(2,6)$, $G{b_{G}^{T}}(45^{\circ },0.5,4,0.5)$,$corr_{G}^{OD}(2)$,	$I_{mn}^{I}(2,5)$, $Y_{rg}^{I}(2,5)$, $Pr_{Rmax(1)}^{N}$, $Gb_{G}^{OD}(135^{\circ },4,2,0.5)$,
	$IM_{1R}^{I}(2)$ $Gb_{G}^{OD}(45^{\circ },0.5,4,0.5)$, $G{b_{G}^{I}}(0^{\circ },0.5,8,0.33)$,	$Gb_{R}^{OD}(0^{\circ },1,2,3),H_{sumR(7)}^{S}$, $G{b_{R}^{T}}(45^{\circ },4,16,2)$,
	$H_{diffR(6)}^{S}$, $G{b_{R}^{I}}(90^{\circ },4,16,1)$, $IM_{1R}^{T}(2)$,	$Gb_{G}^{OD}(90^{\circ },2,16,3)$, $G{b_{R}^{I}}(135^{\circ },3,4,0.5)$, $H_{sumR(10)}^{I}$,
	$Gb_{R}^{OD}(0^{\circ },0.33,4,1)$	$G{b_{R}^{T}}(0^{\circ },0.5,2,2)$
LDA	$C_{shadeG}^{S}(2)$, $IM_{1R}^{I}(2)$, $IM_{2R}^{S}(2)$, $C_{shadeG(10)}^{S}$,	$I_{mn}^{S}(3,7)$, *R* *G* ^*I*^(2,5), $E_{R(3)}^{S}$,$G{b_{G}^{N}}(0^{\circ },3,2,1)$, $\mu _{sumG(1)}^{N}$,
	$IM_{1G}^{OD}(16)$, $IM_{1G(4)}^{OD}$, $Gb_{R}^{OD}(135^{\circ },1,8,3)$	$E_{R(1)}^{S}$, $IM_{2R(4)}^{I}$, $H_{diffR(10)}^{N}$, $\mu _{sumG(2)}^{N}$, $C_{promG(1)}^{N}$,
		$H_{diffG(2)}^{I}$
QDA	$E_{R(7)}^{S}$, $G{b_{G}^{I}}(45^{\circ },1,4,1)$, $corr_{R(4)}^{I}$, *B* *Y* ^*OD*^(4,8),	$I_{mn}^{S}(3,7)$, *R* *G* ^*I*^(2,6), $G{b_{R}^{T}}(0^{\circ },0.5,4,0.33)$, $H_{diffG}^{S}(8)$,
	$H_{sumR(6)}^{S}$, $G{b_{R}^{N}}(0^{\circ },1,4,0.5)$, $G{b_{G}^{S}}(135^{\circ },2,2,1)$,	$G{b_{R}^{T}}(45^{\circ },4,2,1)$, $G{b_{R}^{N}}(45^{\circ },4,2,1)$, $corr_{R(1)}^{T}$, ${E_{G}^{I}}(8)$,
	$C_{shadeG(9)}^{T}$, $G{b_{G}^{I}}(0^{\circ },3,16,1)$	$con_{G}^{N(9)}$, $\mathcal {N}_{xxG}^{N}(8)$
**Whole Image Features**		
AUC	*I* *M*_1*G*_(2), *I* *M*_1*R*_(8), *C*_*promG*(10)_, *G* *b*_*R*_(135^∘^,0.33,8,0.5),	*I* *M*_1*G*_(8), ${\psi ^{H}}_{RAvg}(db3)$, ${\psi ^{H}}_{RAvg}(rbio3.7)$, *I* *M*_1*G*_(1),
	*C*_*promR*(6)_, *C*_*promG*(6)_	*c* *o* *n*_*G*_(8),*H*_*G*(4)_, *H*_*diffG*_(4), *G* *b*_*G*_(135^∘^,0.5,16,0.33),
		*I* *M*_1*R*_(16), *G* *b*_*G*_(90^∘^,2,8,0.33)
LDA	*I* *M*_1*G*(8)_, *G* *b*_*R*_(45^∘^,4,2,1), *G* *b*_*G*_(135^∘^,2,16,1), *E*_*R*(9)_,	*I* *M*_1*G*_(8), *R* *G*(4,8), *I* *M*_1*G*(8)_, *a* *c* *o* *r* *r*_*G*(1)_, ${\psi ^{D}}_{GAvg}(db3)$,
	*h* *o* *m* *o* *m*_*R*(8)_	*R* *G*(3,7), *σ*_*sosG*_(4), *G* *b*_*G*_(90^∘^,4,4,0.5),*G* *b*_*G*_(45^∘^,2,4,0.5),
		*R* *G*(3,7), *σ*_*sosG*(3)_, *E*_*R*_(8)
QDA	*I* *M*_1*G*(8)_, *I* *D* *N*_*G*_(16),*G* *b*_*R*_(135^∘^,0.33,8,0.5), *d* *i* *s* *s*_*G*_(4),	*I* *M*_1*G*_(8), *H*_*diffG*(2)_, *h* *o* *m* *o* *m*_*G*(3)_, *c* *o* *n*_*G*(9)_
	${\psi ^{D}}_{REnergy}(haar)$	

**Table 4:** Input parameters for the classifiers

Classifier type	Parameter values
Twin SVM	C1=6, C2 = 6.14, *𝜖*_1_=0.2, *𝜖*_2_=0.1
Linear SVM	*C*=4
Polyniomial SVM	Γ=0.9, *d*=1, *C*=1
RBF SVM	Γ=0.05, *C*=4
Sigmoid SVM	Γ=0.05, *c* *o* *e* *f* *f*0=1, *C*=1

#### Classifier setting

On selected regional image features, we have constructed the binary classifier for glaucoma classification using Support Vector Machines (SVM) [42]. In recent studies [43], non-parallel SVM has performed better compared to traditional SVM methods. In traditional SVM, two parallel planes are generated such that each plane is as far apart as possible however in non-parallel SVM, the condition of parallelism is dropped. Among non-parallel SVM, Twin SVM has performed better compared to its other counterparts [44]. Mathematically, the Twin SVM is constructed by solving two quadratic programming problems

15 $$ \begin{array}{lllll} \min_{w_{s1},b_{s1},q_{s}} \frac{1}{2} (X_{s1}w_{s1}\,+\,\epsilon_{1}b_{s1})^{T}(\!X_{s1}w_{s1}\,+\,\epsilon_{2}b_{s1})+C_{1}\epsilon_{1}q\\ s.t. -(X_{s2}w_{s1}+\epsilon_{2}b_{s1})+q \geq \epsilon_{2}, q\geq0 \end{array} $$

16 $$ \begin{array}{llll} \min_{w_{s2},b_{s2},q_{s}} \frac{1}{2} (X_{s2}w_{s2}\,+\,\epsilon_{1}b_{s2})^{T}(X_{s2}w_{s2}\,+\,\epsilon_{2}b_{s2})\,+\,C_{2}\epsilon_{1}q\\ s.t. -(X_{s1}w_{s2}+\epsilon_{1}b_{s2})+q \geq \epsilon_{1}, q\geq0 \end{array} $$

The performance of Twin SVM has been compared with traditional SVM. The traditional SVM classifier can be expressed as:

17 $$ \begin{array}{llll} \max_{\alpha \geq 0} \sum\limits_{i} \alpha_{i} - \frac{1}{2}\sum\limits_{j,k}\alpha_{j} \alpha_{k} y_{j} y_{k} k(x_{j},x_{k}) \\ \text{subject to} 0\leq \alpha_{i} \leq C \text{ and} \sum\limits_{i} \alpha_{i} y_{i} = 0 \end{array} $$

where *C* is the penalty term. *k*(*x*_*i*_,*x*) represents the kernel function. In linear SVM case, *k*(*x*_*j*_,*x*_*k*_) = *x*_*j*_.*x*_*k*_. The kernel function in Eq. 17 can be replaced for developing non-linear SVM classifier such as Radial Based Function, polynomial and sigmoid SVM. The *k*(*x*_*j*_,*x*_*k*_) in Eq. 17 is replaced with gaussian kernel mentioned as: *k*(*x*_*i*_,*x*)= exp(−Γ||*x*_*i*_−*x*||^2^). In polynomial function the *k*(*x*_*j*_,*x*_*k*_)=(Γ*x*_*j*_.*x*_*k*_)^*d*^ and in sigmoid SVM *k*(*x*_*j*_,*x*_*k*_) = *t* *a* *n* *h*(Γ*x*_*j*_.*x*_*k*_ + *c* *o* *e* *f* *f*0), where *c* *o* *e* *f* *f*0 is sigmoid coefficient. We have tested different paremeters on libsvm [42] on C-SVC for each kernel function parameters and cost value. We have tested different parameter values for these classifiers and the values for which the respective SVM classifier performed the best in both fundus and SLO images have been shown in Table 4.

Apart from SVM classifiers, we have also compared the peformance with LDA and QDA as they have also been involved in the feature selection process

## Experimental evaluation and discussion

### Evaluation metrics

For optic disc segmentation performance, we have Dice Coefficient [45] as an evaluation measurement, which is the degree of overlap of two regions. It is defined as:

18 $$ D(A,B) = \frac {2 |A \cap B|}{|A \cup B|}, $$

where *A* and *B* are the segmented regions surrounded by model boundary and annotations from the ophthalmologists respectively, ∩ denotes the intersection and ∪ denotes the union. Its value varies between 0 and 1 where a higher value, indicates an increased degree of overlap. Apart from that we have adopted standard evaluation metrics using accuracy (*A* *c* *c*), sensitivity (*S* *n* or true positive rate) and specificity (*S* *p* or false positive rate) described as follows:

19 $$ \begin{array}{llll} Acc = \frac{TN+TP}{TN+TP+FN+FP} \\ Sn = \frac{TP}{TP+FN} \\ Sp = \frac{TN}{TN+FP} \end{array} $$

where *T* *P*,*T* *N*,*F* *P* and *FN* are true positives, true negatives, false positives and false negatives respectively. The significance of the improvement of the classification accuracy has been evaluated by McNemar’s test [46]. The McNemar’s test can be used to compare classification results across different methods and can generate Chi-squared value as:

20 $$ {\chi}^{2} = \frac{(|c1_{err}-c2_{err}|-1)^{2}}{c1_{err}+c2_{err}} $$

where *c*1_*err*_ and *c*2_*err*_ are the number of images misclassified by different methods. We have compared the classification performance of RIFM model to the geometric methods as well as non-geometric methods. The Chi-squared value generated is then converted to *p*_*value*_ for testing statistical significance of the improvement. The test is considered statistically significant if the *p*_*value*_ is below certain value. Typical standard values are 0.1, 0.05 and 0.01 (*χ* ^2^ = 2.706,3.841 and 6.635 respectively).

### Accuracy comparison with the state-of-the-art approaches

We have conducted experimental evaluation on both fundus and SLO image datasets from three aspects:

1. Optic disc segmentation accuracy performance.
2. Accuracy performance based on different classification algorithms and feature selection methods.
3. Accuracy performance comparison with either geometric or non-geometric methods.

The image datasets used for the evaluation are described in “Datasets used for experimentation”, consisting of a representative and heterogeneous image dataset including both fundus and SLO images totalling 189 images; 124 from fundus dataset and 65 from SLO dataset. Each of the fundus and SLO dataset has been split into cross-validation sets and the test sets. In the cross-validation sets, N-fold cross validation [12] has been performed for classification model validation. The essence of n-fold cross validation is to randomly divide a dataset into n equal sized subsets and of the n subsets, a single subset is retained as the validation data for testing the model, and the remaining n-1 subsets are used as training data. The cross-validation process is then repeated n times (the folds), with each of the n subsamples used exactly once as the validation data. The cross-validation accuracy has been determined after training the classifier on n-1 subsets and testing on the nth subset. This has been performed for each subset in the cross-validation set. The cross-validation sets for classifier training are different from that of feature selection process. The accuracy on the test sets for each dataset are then calculated after training the classifier on the images of cross-validation sets of the respective dataset. Additionally, to address dataset imbalance, the Ensemble Random Under Sampling (ERUS) is used, in which useful samples can be selected for learning classifiers [47].

#### Optic disc segmentation accuracy performance

**Fig. 10:**
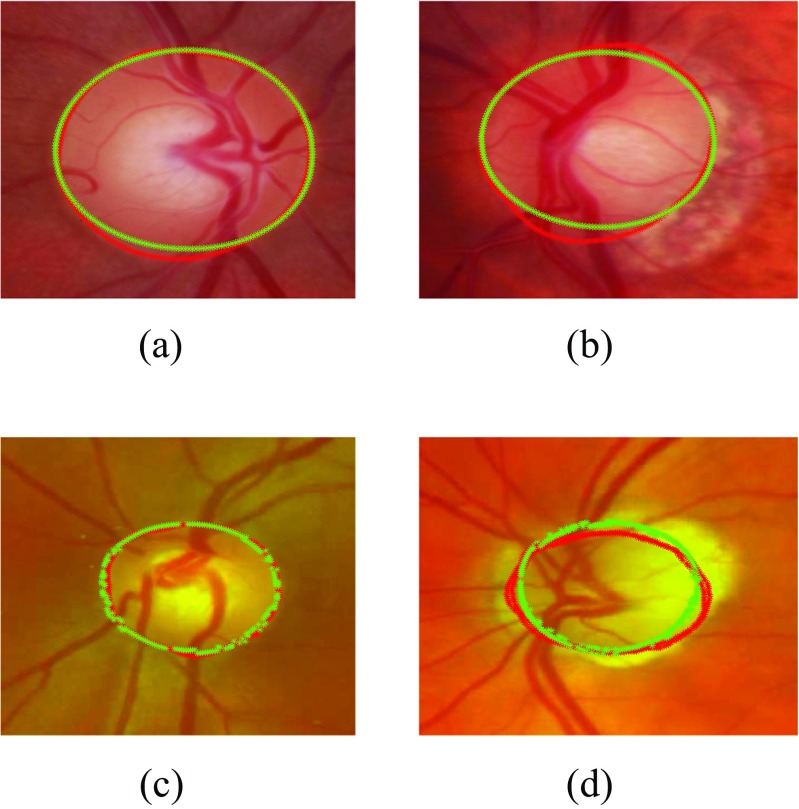
Examples of optic disc segmentation using proposed approach **a,b** are examples from RIM-ONE and **c,d** are examples from SLO images. The *red* outline shows the original annotation around optic disc whereas the *green* outline shows the automatic annotations from proposed approach

**Fig. 11:**
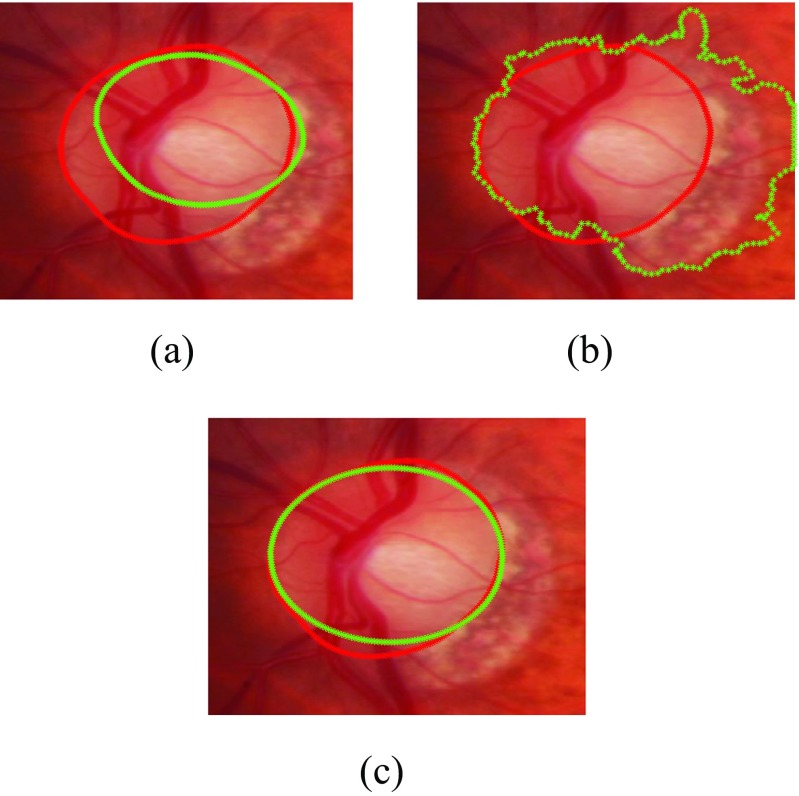
Comparison of optic disc segmentation of proposed approach with previous methods **a** Active Shape Model [48], **b** Chan-Vese [49] and **c** the proposed approach

We have compared our segmentation methods with clinical annotations and existing models such as Active Shape Model [48], Chan-Vese [49]. The experimental results are shown in Figs. 10 and 11 and our method outperforms the existing methods. The mean and standard deviation of Dice Coefficients of our previous approach [20] and proposed approach has been evaluated on both RIM-ONE and SLO datasets with respect to both healthy and glaucomatous images as shown in Table 5. Also some of the examples of optic disc segmentation compared to clinical annotations has been shown in Fig. 10. The visual results show that segmentation accuracy is quite comparable to clinical annotation; especially in the right column which represent the examples of glaucomatous optic disc with PPA.

**Table 5:** Accuracy comparison of the proposed optic disc segmentation approach with our previous approach

	RIM-ONE			SLO images		
	Normal	Glaucoma	Both	Normal	Glaucoma	Both
**The Proposed Approach**	**0** **.** **9** **5** ***±*** **0** **.** **0** **3**	**0** **.** **9** **2** ***±*** **0** **.** **0** **7**	**0** **.** **9** **4** ***±*** **0** **.** **0** **5**	**0** **.** **9** **1** ***±*** **0** **.** **0** **7**	**0** **.** **8** **9** ***±*** **0** **.** **0** **7**	**0** **.** **9** **0** ***±*** **0** **.** **0** **7**
Active Shape Model	0.91 ± 0.06	0.87 ± 0.09	0.89 ± 0.06	0.82 ± 0.10	0.80 ± 0.08	0.81 ± 0.09
Chan-Vese Model	0.92 ± 0.06	0.84 ± 0.12	0.89 ± 0.07	0.85 ± 0.10	0.82 ± 0.12	0.84 ± 0.10

#### Accuracy comparison based on different classification algorithms and feature selection methods

The performance of regional features selected under the proposed approach compared with other regional feature selection methods across different classifiers have been presented in Table 6 for cross-validation sets and in Table 7 for the test-sets. According to the results, the feature sets selected by AUC maximization have higher accuracy on both cross-validation sets and the test sets compared to the ones selected by maximization of linear and quadratic classification accuracy. The results also show that dropping the parallelization condition from the SVM can have marginal improvement in terms of classification accuracy; like in case of Twin SVM. Moreover, classifier with linear specifications i.e. Linear SVM and LDA have performed significantly better compared to other non-linear counterparts. The performance of Polynomial SVM is comparable to Linear SVM however, it has achieved this accuracy at degree *d*=1 which is the special case of linear classification. The performance of the classifiers on cross-validation sets and the test sets have been combined and detailed in Table 8. In Table 8, we have compared the classifier performance with respect to sensitivity and specificity along with classification accuracy. We have identified the best results of each classifier across different feature sets mentioned in Table 6. For example, Twin SVM has the best results on wrapper-AUC or RBF-SVM has the best results on wrapper-QDA so they are the best feature set for the respective classifiers. The results show that the non-linear classifiers such as RBF-SVM and QDA have high false negatives compared to their linear counterparts which have resulted the depreciation in their performance. The Twin SVM classifier has achieved the accuracy of 94.4 % on fundus images and 93.9 % on SLO image dataset.

**Table 6:** Comparison of classification accuracies across different feature selection methods in cross-validation set

Classifier	RIMONE			SLO images		
	wrap-AUC	wrap-LDA	wrap-QDA	wrap-AUC	wrap-LDA	wrap-QDA
Twin SVM	96.3 %	90.0 %	78.8 %	94.1 %	84.3 %	78.4 %
Linear SVM	95.0 %	90.0 %	81.3 %	94.1 %	84.3 %	78.4 %
Polynomial SVM	95.0 %	90.0 %	81.3 %	94.1 %	84.3 %	78.4 %
RBF SVM	90.0 %	87.5 %	82.5 %	82.3 %	78.4 %	82.3 %
Sigmoid SVM	78.8 %	92.5 %	77.5 %	78.4 %	74.5 %	78.4 %
LDA	95.0 %	88.8 %	80.0 %	90.5 %	82.4 %	78.4 %
QDA	85.0 %	81.3 %	86.3 %	78.4 %	68.6 %	82.4 %

**Table 7:** Comparison of classification accuracies across different feature selection methods in test set

Classifier	RIMONE			SLO images		
	wrap-AUC	wrap-LDA	wrap-QDA	wrap-AUC	wrap-LDA	wrap-QDA
Twin SVM	90.9 %	86.4 %	81.8 %	85.7 %	78.6 %	64.3 %
Linear SVM	90.9 %	88.6 %	81.8 %	78.6 %	71.4 %	64.3 %
Polynomial SVM	90.9 %	88.6 %	81.8 %	78.6 %	71.4 %	64.3 %
RBF SVM	88.6 %	86.4 %	97.7 %	78.6 %	57.1 %	78.6 %
Sigmoid SVM	84.1 %	86.4 %	86.4 %	64.3 %	28.6 %	28.6 %
LDA	88.6 %	88.6 %	79.5 %	71.4 %	71.4 %	64.3 %
QDA	86.4 %	88.6 %	90.9 %	64.3 %	35.7 %	74.1 %

**Table 8:** Comparison of sensitivity, specificity and accuracy across different classifiers

Classifier	RIMONE								SLO images					
	TP	FN	TN	FP	Sn	Sp	Acc	TP	FN	TN	FP	Sn	Sp	Acc
**Twin SVM**	**36**	**3**	**81**	**4**	**92.3 %**	**95.3 %**	**94.4 %**	**17**	**2**	**43**	**4**	**89.5 %**	**93.5 %**	**93.9 %**
Linear SVM	36	3	80	5	92.3 %	94.1 %	93.5 %	17	2	42	4	89.5 %	91.3 %	90.8 %
Polynomial SVM	36	3	80	5	92.3 %	94.1 %	93.5 %	18	1	41	5	94.7 %	89.1 %	90.8 %
RBF-SVM	31	8	80	5	79.5 %	94.1 %	89.5 %	14	5	39	7	73.7 %	86.7 %	81.5 %
Sigmoid SVM	32	7	80	5	82.1 %	94.1 %	90.3 %	15	4	34	12	78.9 %	73.9 %	75.4 %
LDA	36	3	79	6	92.3 %	92.9 %	92.7 %	14	5	38	8	73.7 %	82.6 %	80.0 %
QDA	30	9	78	7	76.9 %	91.8 %	87.1 %	10	9	42	4	52.6 %	91.3 %	80.0 %

#### Accuracy comparison with either geometric or non-geometric based methods

**Fig. 12:**
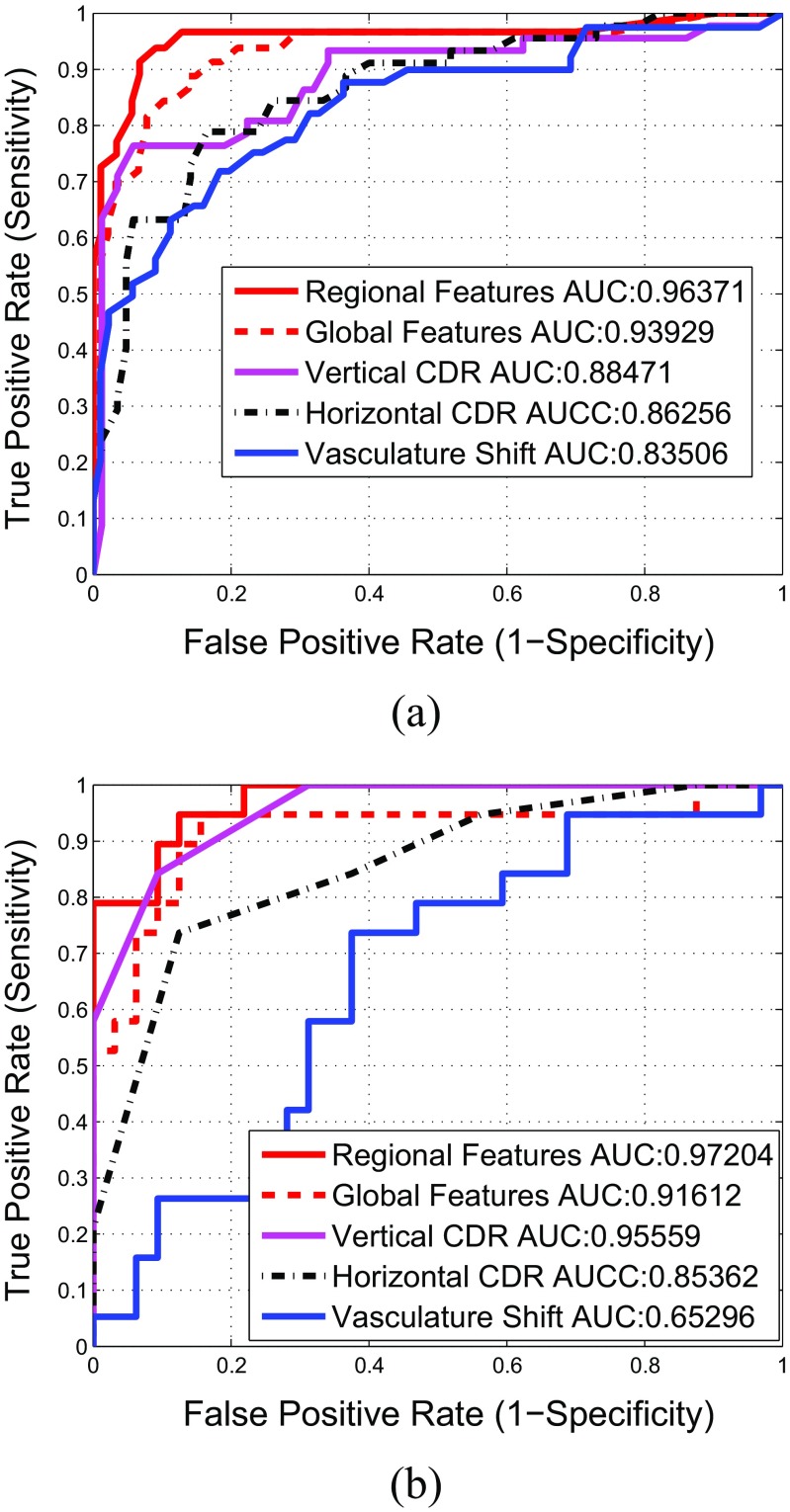
Comparison of Receiver Operating Characteristics of different feature sets mentioned in Table 9

To validate our proposed method, we have compared the performance of RIFM with 1) geometrical based clinical indicators on glaucoma such as vertical and horizontal CDRs, vasculature shift, and 2) the existing methods using non-geometrical global features [15, 18, 19]. In case of geometrical indicators, both vertical as well as horizontal CDR has been clinically annotated for both fundus and SLO images whereas vasculature shift has been determined automatically using the method mentioned in [50]. The cutoff value for both CDRs is set to 0.55. In case of non-geometrical features, we have calculated global image features under the same procedure as in case of regional features except that they are calculated for whole optic disc cropped image. Like regional features, we have constructed a global image feature model under Twin SVM on the features selected by wrapper-AUC approach under the classifier parameters where global features performed the best. The performance comparison is shown with respect to ROC curves in Fig. 12 and has been quantified in Table 9. Moreover the significance of classification improvement of RIFM model has also been compared with other geometric and non-geometric based methods by McNemar’s test (4). The results show that in case of both fundus and SLO dataset, the RIFM model shows significant improvement in glaucoma classification in most of the geometric and non-geometric based methods (*p*_*value*_≤0.05, 0.10). In case of clinically annotated vertical CDR and non-geometric textural features, the results can show improvement at significance level *p*_*value*_≤0.10.

**Table 9:** Accuracy comparison of the proposed RIFM model with either geometric or non-geometric-based methods

	RIMONE								SLO Images							
Features (i.e. geometric or	TP	FN	TN	FP	Sn	Sp	Acc	*p*_*value*_	TP	FN	TN	FP	Sn	Sp	Acc	*p*_*value*_
non-geometric)																
**RIFM**	**36**	**3**	**81**	**4**	**92.3 %**	**95.3 %**	**94.4 %**	**–**	**17**	**2**	**31**	**1**	**89.5 %**	**96.9 %**	**94.1 %**	**–**
Geometric based Methods																
Geo-metric (Vertical CDR)	29	10	80	5	74.4 %	94.1 %	87.9 %	<0.10	16	3	29	3	84.2 %	90.6 %	88.2 %	=0.28
Geo-metric (Horizontal CDR)	26	13	76	9	66.7 %	89.4 %	82.3 %	<0.01	14	5	28	4	73.7 %	87.5 %	82.4 %	<0.10
Geo-metric (Vasculature Shift)	26	13	75	10	66.7 %	88.2 %	81.5 %	<0.01	14	5	20	12	73.7 %	62.5 %	66.7 %	<0.001
Non-geometric based Methods																
Global Features (Mix)	35	4	74	11	89.7 %	87.1 %	87.9 %	<0.10	13	6	28	4	68.4 %	87.5 %	80.4 %	<0.10
Textural Features (Variable Offset) [18, 19]	30	9	71	14	76.9 %	83.5 %	81.5 %	<0.01	11	8	18	12	57.9 %	56.2 %	56.9 %	<0.001
Textural Features (Variable Scale) [18, 19]	35	4	74	11	89.7 %	87.1 %	87.9 %	<0.10	12	7	21	11	63.2 %	65.6 %	64.7 %	<0.005
Textural Features (Scale + Offset) [18, 19]	35	4	74	11	89.7 %	87.1 %	87.9 %	<0.10	13	6	28	4	68.4 %	87.5 %	80.4 %	<0.10
Higher Order Spectra Features [19]	34	5	74	11	87.2 %	87.1 %	87.1 %	<0.05	12	7	24	8	63.2 %	75.0 %	70.6 %	<0.01
Gabor Features [51]	34	5	75	10	87.2 %	88.2 %	87.9 %	<0.10	11	8	24	8	57.9 %	75.0 %	68.6 %	<0.01
Wavelet Features [15]	31	8	65	20	79.5 %	76.5 %	77.4 %	<0.001	11	8	24	8	57.9 %	75.0 %	68.6 %	<0.01
Gaussian Features	32	7	67	18	82.1 %	78.8 %	79.8 %	<0.01	10	9	26	6	52.6 %	81.3 %	70.6 %	<0.05
Dyadic Gaussian Features	28	11	75	10	71.8 %	88.2 %	83.1 %	<0.05	10	9	26	6	52.6 %	81.3 %	70.6 %	<0.05

#### Discussion

Based on our experimental evaluation, the proposed method after automatically locating and segmenting the optic disc as well as dividing the optic disc cropped image into different regions extract the regional features reflecting pixel appearance such as textural properties, frequency based information, gradient features etc. In this way the geometrical properties due to large cup size in glaucoma can be quantified and accommodated with textural changes within optic disc boundary. Moreover, the model can also accommodate the non-geometric based features from different regions around optic disc boundary. The feature selection and classification results suggests that different types of features for different regions of optic disc and its surroundings can result in better classification performance. The significance results shows that our proposed RIFM model has performed significantly better compared to the geometrical methods based on segmentation of glaucoma associated anatomical structures for determination of clinical indicators of either CDR or vasculature shift as well as non-geometrical methods based on global image feature model. This further validates our idea that if both geometrical and non-geometrical indications are combined together, this can significantly increase the glaucoma classification performance.

## Conclusion

In this paper, we have proposed the novel computer-aided approach: *Regional Image Features Model* (RIFM) which can extract both geometric and non-geometric properties from an image and automatically perform classification between normal and glaucoma images on the basis of regional image information. The proposed method automatically localises and segments the optic disc, divides the optic disc surroundings into different regions and performs glaucoma classification on the basis of image-based information of different regions. The novelties of the work include 1) a new accurate method of automatic optic disc localisation; 2) a new accurate method of optic disc segmentation; 3) a new RIFM on extraction of both geometric and non-geometric properties from different regions of optic disc and its surroundings for classification between normal and glaucoma images.

The performance of our proposed RIFM model has been compared across different feature sets, classifiers and previous approaches and has been evaluated on on both fundus and SLO image datasets. The experimental evaluation result shows our approach outperforms existing approaches using either geometric or non-geometric approaches. The classification accuracy on fundus and SLO images is 94.4 % and 93.9 % respectively. The results validate our hypothesis of combining both geometrical and non-geometrical indications since they are significantly better compared to methods which are based on either geometrical or non-geometrical indications.

Further research is needed to test the model on datasets composed of healthy as well as various stages of glaucoma. Additionally, because the most common clinical indicator for glaucoma detection is to measure CDR value (based on manual approaches), we will further develop the proposed RIFM approach for automated CDR measurement.

## Appendix A

**Table 10:** Textural features extracted using GLCM

Feature name	Equation	Definition
Autocorrelation	$ acorr = \sum \limits _{i}\sum \limits _{j} ij{p(i,j)}$	Linear dependence in GLCM between same index
Cluster Shade	$ C_{shade} = \sum \limits _{i}\sum \limits _{j}(i+j-\mu _{x}-\mu _{y})^{3} p(i,j) $	Measure of skewness or non-symmetry
Cluster Prominence	$C_{prom} = \sum \limits _{i}\sum \limits _{j}(i+j-\mu _{x}-\mu _{y})^{4} p(i,j) $	Show peak in GLCM around the mean for non-symmetry
Contrast	$ con = {\sum \limits _{i=1}^{N_{g}}\sum \limits _{j=1}^{N_{g}}|i-j|^{2}p(i,j)} $	Local variations to show the texture fineness.
Correlation	$ corr = \frac {\sum \limits _{i}\sum \limits _{j} (ij)p(i,j)-\mu _{x}\mu _{y}}{\sigma _{x}\sigma _{y}}$	Linear dependence in GLCM between different index
Difference Entropy	$ H_{diff} = -\sum \limits _{i=0}^{N_{g}-1}p_{x-y}log(p_{x-y}(i))$	Higher weight on higher difference of index entropy value
Dissimilarity	$ diss = \sum \limits _{i}\sum \limits _{j}|i-j|p(i,j)$	Higher weights of GLCM probabilities away from the diagonal
Energy	$ E = \sum \limits _{i}\sum \limits _{j}{p(i,j)}^{2}$	Returns the sum of squared elements in the GLCM
Entropy	$ H = -\sum \limits _{i}\sum \limits _{j}p(i,j)log(p(i,j))$	Texture randomness producing a low value for an irregular GLCM
Homogeneity	$ homom = \sum \limits _{i}\sum \limits _{j}\frac {1}{1+(i-j)^{2}}p(i,j)$	Closeness of the element distribution in GLCM to its diagonal
Information Measures 1	*I* *M*_1_=(1−*e* *x* *p*[−2.0(*H*_*x**y*_−*H*)])^0.5^	Entropy measures
Information Measures 2	$ IM_{2}=\frac {Entropy-H_{xy2}}{\text {MAX}(H_{x},H_{y})}$	Entropy measures
Inverse Difference	$ IDN = \sum \limits _{i} \sum \limits _{j} \frac {p(i,j)}{1+\frac {|i-j|}{N_{g}}}$	Inverse Contrast Normalized
Normalized		
Inverse Difference Moment	$ IDMN = \sum \limits _{i} \sum \limits _{j} \frac {p(i,j)}{1+\frac {(i-j)^{2}}{N_{g}}}$	Homogeneity Normalized
Normalized		
Maximum Probability	$ Pr_{max} = \underset {(x,y)}{\text {MAX}}p(i,j)$	Maximum value of GLCM
Sum average	$ \mu _{sum} = \sum \limits _{i=2}^{2N_{g}}ip_{x+y}(i)$	Higher weights to higher index of marginal GLCM
Sum Entropy	$ H_{sum} = -\sum \limits _{i=2}^{2N_{g}}p_{x+y}log(p_{x+y}(i))$	Higher weight on higher sum of index entropy value
Sum of Squares: Variance	$ \sigma _{sos} = \sum \limits _{i}\sum \limits _{j}(i-\mu )^{2}p(i,j)$	Higher weights that differ from average value of GLCM
Sum of Variance	$ \sigma _{sum} = \sum \limits _{i=2}^{2N_{g}}(i-H_{sum})p_{x+y}(i)$	Higher weights that differ from entropy value of marginal GLCM

(*i*,*j*) represent rows and columns respectively, *N*_*g*_ is number of distinct grey levels in the quantised image, *p*(*i*,*j*) is the element from normalized GLCM matrix *p*_*x*_(*i*) and *p*_*y*_(*j*) are marginal probabilities of matrix obtained by summing rows and columns of GLCM respectively i.e. $p_{x}(i)=\sum \limits _{j=1}^{N_{g}}p(i,j)$, $p_{y}(j)=\sum \limits _{i=1}^{N_{g}}p(i,j)$, $p_{x+y}(k)=\sum \limits _{i=1}^{N_{g}}\sum \limits _{j=1}^{N_{g}}p(i,j), k=i+j-1=1,2,3,....,2N_{g}$ and $p_{x-y}(k)=\sum \limits _{i=1}^{N_{g}}\sum \limits _{j=1}^{N_{g}}p(i,j), k=|i-j|+1=1,....,N_{g}$, *H*_*x*_ and *H*_*y*_ and entropies of *p*_*x*_ and *p*_*y*_ respectively, $H_{xy}=-\sum \limits _{i}\sum \limits _{j}p_{x}(i)p_{y}(j)log(p_{x}(i)p_{y}(j))$, $H_{xy2}=-\sum \limits _{i}\sum \limits _{j}p(i,j)log(p_{x}(i)p_{y}(j))$
